# Development and Characterization of Curcumin-Loaded TPGS/F127/P123 Polymeric Micelles as a Potential Therapy for Colorectal Cancer

**DOI:** 10.3390/ijms25147577

**Published:** 2024-07-10

**Authors:** Rita Cerqueira, Cátia Domingues, Francisco Veiga, Ivana Jarak, Ana Figueiras

**Affiliations:** 1 Laboratory of Drug Development and Technologies, Faculty of Pharmacy, University of Coimbra, 3000-548 Coimbra, Portugal; ritacerqueira2000@gmail.com (R.C.); cdomingues@ff.uc.pt (C.D.); fveiga@ff.uc.pt (F.V.); jarak.ivana@gmail.com (I.J.); 2 REQUIMTE/LAQV, Group of Pharmaceutical Technology, University of Coimbra, 3000-548 Coimbra, Portugal; 3 Institute for Clinical and Biomedical Research (iCBR) Area of Environment Genetics and Oncobiology (CI MAGO), Faculty of Medicine, University of Coimbra, 3000-548 Coimbra, Portugal; 4 Instituto de Investigação e Inovação em Saúde, University of Porto, 4200-135 Porto, Portugal

**Keywords:** colorectal cancer (CRC), curcumin (CUR), polymeric micelles (PMs)

## Abstract

Colorectal cancer (CRC) is the third most prominent cancer worldwide, and the second leading cause of cancer death. Poor outcomes and limitations of current treatments fuel the search for new therapeutic options. Curcumin (CUR) is often presented as a safer alternative for cancer treatment with a staggering number of molecular targets involved in tumor initiation, promotion, and progression. Despite being promising, its therapeutic potential is hindered due to its hydrophobic nature. Hence, the ongoing development of optimal delivery strategies based on nanotechnology, such as polymeric micelles (PMs), to overcome issues in CUR solubilization and delivery to tumor cells. In this sense, this study aimed to optimize the development and stability of CUR-loaded P123:F127:TPGS PMs (PFT:CUR) based on the thin-film approach and evaluate their therapeutic potential in CRC. Overall, the results revealed that the solubility of CUR was improved when room temperature was used to hydrate the film. The PFT–CUR hydrated at room temperature presents an average hydrodynamic diameter of 15.9 ± 0.3 nm with a polydispersity index (PDI) of 0.251 ± 0.103 and a zeta potential of −1.5 ± 1.9 mV, and a 35.083 ± 1.144 encapsulation efficiency (EE%) and 3.217 ± 0.091 drug loading (DL%) were observed. To ensure the stability of the optimized PFT–CUR nanosystems, different lyophilization protocols were tested, the use of 1% of glycine (GLY) being the most promising protocol. Regarding the critical micellar concentration (CMC), it was shown that the cryoprotectant and the lyophilization process could impact it, with an increase from 0.064 mg/mL to 0.119 mg/mL. In vitro results showed greater cytotoxic effects when CUR was encapsulated compared to its free form, yet further analysis revealed the heightened cytotoxicity could be attributed to the system itself. Despite challenges, the developed CUR-loaded PM shows potential as an effective therapeutic agent for CRC. Nonetheless, the system must undergo refinements to enhance drug entrapment as well as improve overall stability.

## 1. Introduction

Colorectal cancer (CRC) is the third most common cancer and the second leading cause of cancer-related deaths globally, with a dismal 5-year survival rate of approximately 14% [1,2,3,4]. This poor prognosis is primarily due to late diagnosis, with 90% of CRC deaths occurring in patients with metastasis [5,6,7,8]. Despite advances in the approved therapeutic schemes, reported off-target effects that lead to adverse events have limited their effectiveness, particularly in metastatic cancer [3,4,6,9], which may contribute to patients relapsing [4,8,10,11]. Therefore, the identification of new therapeutic agents and target therapies capable of inducing tumor-specific cytotoxicity can minimize adverse effects, and overcome some of the reported challenges [8]. 

Curcumin (CUR) is an active molecule found in the rhizomes of Curcuma longa [12,13,14]. Its application for medicinal purposes roots back traditional practices and continues inspiring researchers due to its diversity of molecular targets and favorable therapeutic index, particularly for its anti-inflammatory, antioxidant, antidiabetic, antibacterial, antifungal, antiproliferative, anticancer, and hepatoprotective effects [15,16,17]. CUR’s vast range of activities can be attributed to its ability to modulate a variety of signal transduction pathways involved in regulatory processes, such as inflammation, immunity, cell cycle, apoptosis, autophagy, and cell metabolism [4,8,9,18,19,20,21,22]. 

Natural compounds, such as CUR, can be a safer alternative for cancer patients due to their lack of toxic effects in humans [8,23]. Despite CUR’s promising therapeutic effects, this polyphenol has very low aqueous solubility, and poor bioavailability in humans, limiting its efficacy, particularly in cancer treatment [12,14,15,24]. Furthermore, issues regarding the instability of CUR, namely an alkaline pH, contribute to its rapid degradation [14,15,23,24,25,26,27,28]. Hence, different strategies have been studied to enhance CUR delivery, namely using nanoformulations. In fact, the loading of CUR into nanocarriers has demonstrated a target delivery into the cancer site or metastatic tissue, exhibiting a higher therapeutic effect, by the enhancement of its biodistribution and bioavailability [12,14,29]. A wide range of CUR nanoparticles (NPs) are currently being investigated for CRC [26,28,30,31,32,33,34]. In particular, biodegradable polymeric NPs and polymeric micelles (PMs) appear to have the most advantageous pharmacokinetic properties [14]. 

PMs (10–100 nm) composed of amphiphilic block copolymers, like poloxamers, can form thermodynamically stable micelles in aqueous solutions [35,36,37,38]. Poloxamers, such as Pluronic^®^, are popular building blocks for engineering nanotechnology-based drug delivery systems (nano-DDSs). They represent a particular class of synthetic non-ionic amphiphilic copolymers, composed of a central hydrophobic chain of poly(propylene oxide) (PPO) and two hydrophilic chains of poly(ethylene oxide) (PEO); the result is a PEO–PPO–PEO triblock self-organizing amphiphilic structure [35,36,38,39]. PMs with Pluronic F127^®^ have extended and well-hydrated outer shells of PEO (Table 1), which rapidly leads to a stable structure and low critical micellar concentration (CMC), ideal for intravenous administration [36,40,41]. On the other hand, Pluronic P123^®^ has a longer PPO chain (Table 1) and has a higher capacity to solubilize hydrophobic drugs [36,42]. Furthermore, some authors defend the cytostatic action of Pluronic P123^®^ micelles, particularly through adenosine triphosphate (ATP) consumption in multidrug-resistant cancer cells, which inhibits efflux P-glycoprotein [42,43,44]. D-ɑ-tocopheryl polyethylene glycol succinate (TPGS) is an FDA-approved pharmaceutical adjuvant, composed of a hydrophobic vitamin E part that facilitates the solubilization of poorly water-soluble compounds, such as CUR, and a hydrophilic PEG chain [45]. Studies have shown TPGS anticancer activity can be attributed to its high selective cytotoxic effect [45]. TPGS has been associated with a potent apoptotic effect via the mitochondrial pathway and reduction in proliferation by cell cycle arrest on multiple cancer cell lines [45,46,47,48]. Overall, Pluronic P123^®^/Pluronic F127^®^/TPGS mixed micelles can be used to enhance solubility and biocompatibility of hydrophobic drugs. The appeal behind this construction lies in the capacity of Pluronic P123^®^ and TPGS to successfully incorporate hydrophobic drugs, and the stabilizing role of Pluronic F127^®^.

The aim of this study is to develop and characterize CUR-loaded P123:F127:TPGS polymeric micelles (PFT:CUR) and identify the appropriate nanosystems based on their physicochemical properties and stability. Various techniques, such as lyophilization, will be explored to optimize the nano-drug delivery system (nano-DDS). Additionally, the in vitro cytotoxic effect of the optimized PFT:CUR will be evaluated on a human CRC adenocarcinoma cell line (LoVo) to assess the therapeutic potential of this new therapeutic option, while also addressing the necessity to improve poor outcomes and limitations of current treatments for this type of cancer.

## 2. Results

### 2.1. Development and Characterization of P123:F127:TPGS Polymeric Micelles

PFT micelles loaded with CUR were initially produced by the conventional thin-film method using acetonitrile (ACN) as solvent, with an optimized procedure for the hydration step defined as methods A to B, as recorded in figure in Section 4.2.1. Then, hydration time was extended (conditions C, D, and E). 

Initial characterization studies (n = 3) of empty micelles (PFT:A) prepared using different polymer ratios (1:1:1, 2:1:1, and 1:2:1) were conducted. The 2:1:1 ratio had a smaller size (<25 nm) and lower PDI (<0.3). A small size and uniform population is relevant for an effective delivery to cancer cells [11]. Therefore, a 2:1:1 ratio was proven to be adequate for micelle formation. In addition, this ratio had been previously used successfully to build PMs for CUR delivery [49].

#### 2.1.1. Particle Size, Polydispersity Index, and Zeta Potential

In an initial analysis, it was verified that incorporating CUR into the PMs had no significant impact on the particle size distribution. However, PMs hydrated without thermal processing (at room temperature (RT)), condition B, successfully achieved a higher entrapment of CUR in the hydrophobic core, exhibited by the improvement of encapsulation efficiency percentage (EE%) and drug loading percentage (DL%) values, from 18.197% to 35.083% and from 1.880% to 3.217%, respectively (Table 2). Additionally, both empty P123:F127:TPGS polymeric micelles (PFTs) and loaded PMs prepared under condition B exhibited a smaller size (15–16 nm), with a narrow distribution pattern, and a neutral zeta potential (ZP) (Table 2), compared to PFT:A and PFT:CUR:A. Possibly, we can anticipate a deeper penetration from the vascular structures into the tumor tissue due to a reduction in the size with PFT:CUR:B. The rationale behind this conclusion also stems from the higher accumulation rate in the tumor site and an extended systemic circulation time achieved with smaller nanoparticles [50,51]. A neutral ZP is also preferable to avoid recognition by RES and prolong blood circulation [52]. Studies have shown that neutral (±10 mV) nanoparticles travelled up to three times more distance than charged NPs, and were distributed more homogeneously within tissue [53,54]. Overall, a smaller size and neutral ZP are desirable to leverage the EPR effect [53].

Likewise, Chuacharoen (2019) also observed an increase in size and polydispersity index (PDI) when CUR nano-DDSs were submitted to thermal processing (63 °C for 30 min and 95 °C for 10 min) [55]. A possible explanation for the observed differences lies in the poor thermostability of CUR. In a few studies, a fast degradation rate of free CUR [56,57] and CUR in nanosystems [56,58] was reported when the temperature was increased. Another possible explanation for the obtained results is the relation between temperature increase and dehydration of the PEO region [55,59]. Other studies have correlated the temperature increase with a growth in particle size, as observed with PFT:CUR:A [60]. 

#### 2.1.2. Quantification of Curcumin Using UV—vis Spectroscopy

The quantification of CUR was performed using the ultraviolet–visible spectroscopy (UV—vis) method based on an adapted reported protocol [49]. After, a series of standard CUR concentrations prepared in 90% ethanol (EtOH) were fitted in a calibration curve (n = 4), and the resulting linear regression (y = 0.1566x + 0.0058; R^2^ = 0.9997) was used to extrapolate the concentration of the compound in the analyzed samples (Figure S2). Adequate linearity was shown in standard CUR concentrations ranging from 0.05 μg/mL to 2.5 μg/mL, with acceptable SD (±2 SD). 

#### 2.1.3. Drug Loading (DL%) and Encapsulation Efficiency (EE%) Percentage Analysis by UV—vis Spectroscopy

The drug load determines the availability of the active substance at the site of action, which tends to be low (<25%) for hydrophobic compounds. In this case, the DL was expected to be approximately 9% (weight of CUR = 40 mg; total weight of PM = 440 mg). However, the obtained values ranged from 2–3% (Table 2 and Table S1). The average EE% ranged from 20–35%. Micellization occurring at RT made CUR more easily loaded into the hydrophobic core. CUR’s low thermostability and heat-related degradation were probable reasons that explain the aforementioned observations. Although hydrating the PMs at RT results in higher EE% and DL% values, these values remained relatively low when compared to what can be found in the literature [49]. This issue could be attributed to CUR’s poor solubility and instability.

Meanwhile, further strategies to increase EE% and DL% percentages should be considered. Strengthening the interaction between the CUR and the core-forming polymers could minimize the amount of undissolved CUR and potentiate drug retention [61]. A possible strategy would be to increase the TPGS ratio, as this would lead to a bulkier inner core, facilitating the retention of CUR inside the micelle core. Increasing the amount of encapsulated CUR could also result in a stronger hydrophobic bond, thus improving the kinetic stability of PMs [61]. Additionally, studies have found that a more hydrophobic core can further protect CUR against oxidative degradation [62]. On the other hand, another often used strategy to enhance the solubility of lipophilic molecules is the formation of curcumin/cyclodextrin (CD) complexes by forming host/guest supramolecules that can be incorporated inside the hydrophobic core [24,63,64]. In addition, an alternative method to improve drug load of lipophilic molecules is the incorporation of polymer-drug conjugates [65,66]. In conclusion, the temperature for PM preparation, the polymer concentration, and the polymer–drug interactions influence the amount of drug incorporated.

#### 2.1.4. Storage Stability

The PM must preserve its structure to efficiently deliver the entrapped active compound. The hydrophilic shell protects the hydrophobic core from exposure to the aqueous environment and avoids PM disassembly and premature loss of hydrophobic cargos [67]. The results in Table 3 reveal a steep escalation of particle size and PDI upon storage at RT for 1 month, with relevant deviation between similar batches. This increase is most likely explained by structural breakdown and particle aggregation [20,68,69]. The reduction in the magnitude of ZP was possibly attributed to the adsorption of free polymers. This may happen because residual chains of polymers that do not form micelles can form an adsorption layer on the surface of particles [70], which might happen due to free TPGS chains or even free CUR [45,49]. Overall, the results point to PM instability after 30 days at RT, probably due to loss of structural integrity. 

**Figure 1:**
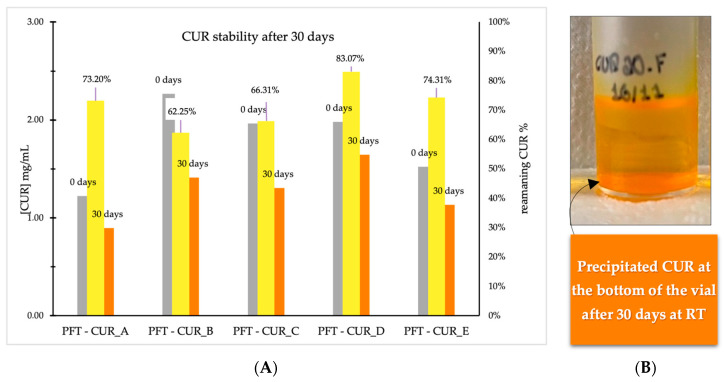
(**A**) Graphical representation of incorporated CUR content (mg/mL) recorded before and after 30 days of storage at RT, and the remaining percentage of CUR. (**B**) Visual aspect of PFT:CUR:B polymeric micelles after 30 days of being produced.

Moreover, Figure 1A demonstrates a decrease in CUR content after 30 days in all groups. This is more significant in PFT:CUR:B, starting from a concentration of CUR of 2.265 mg/mL and dropping to 1.410 mg/mL, revealing a loss in the entrapped CUR of nearly 40%. Coincidently, this group initially had the highest concentration of entrapped CUR. This confirms the hypothesis that the PM gradually aggregates, which fragilizes its structural integrity and leads to a significant loss in the amount of loaded CUR. Consequently, CUR that diffuses from the hydrophobic core precipitates due to its low water solubility (Figure 1B). Interestingly, Degobert (2021) found that PMs with a thinner membrane (1.5–35 nm) are easily disrupted, favoring drug leakage. And, in fact, PFT:CUR PMs were on the smaller side (15–22 nm) [68]. 

From the results of physical and chemical stability studies, it can be concluded that the developed PMs (PFT:CUR) faced critical problems after 1 month in storage at RT and protected from light. Fusion of the PMs and the release of CUR resulting in precipitation and loss of the core-shell structure appear to be the main reasons for the underlying system’s instability. Therefore, storage of formulations at RT might not be the most suitable protocol to address stability.

### 2.2. Development and Characterization of Lyophilized P123:F127:TPGS Polymeric Micelles

**Figure 2:**
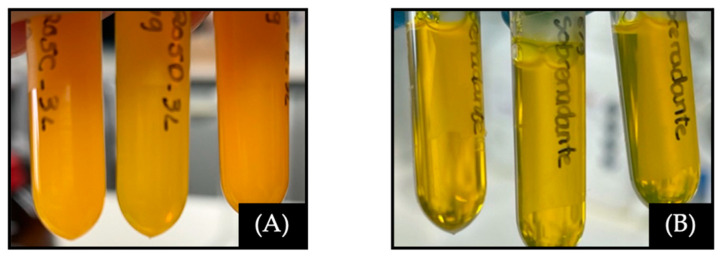
(**A**) PFT:CUR after lyophilization and reconstitution in ultra-pure H2O (pH = 7). (**B**) Same samples after being centrifugated and the supernatant collected.

As discussed previously, PMs after lyophilization should be easily resuspended, present no modification in size distribution, and preserve the amount of loaded drug. Unfortunately, lyophilized samples containing CUR could not be easily re-dispersed into a clear solution (Figure 2A). In a way, PFT:CUR suffered physiochemical alterations. In contrast, blank PMs showed no relevant changes in size and ZP. 

The first approach was to centrifuge samples to sediment the undissolved CUR and collect the dissolved portion (Figure 2B). Afterwards the supernatant’s physicochemical properties were analyzed (Table S2). As expected, there was a notable decrease in the amount of CUR in all samples. Similar studies have reported a loss in drug content after re-hydration, promoted by excessive aggregation, exposure of the hydrophobic regions, and consequently PM breakage [68,71,72]. The increase in negative charge after lyophilization may be due to the rearrangement of free polymers on the micelle surface [73].

#### 2.2.1. Cryoprotectors (Pre-Lyophilization)

In an attempt to improve the freeze-dried powder re-dispersibility, a cryoprotector, more specifically glycine (GLY), was added to the solutions at concentrations of 1% (*w*/*v*). This decision was based on previously reported positive feedback [71]. However, after reconstitution the hydrated powders did not present their original clear appearance.

#### 2.2.2. Re-Constitution (Post-Lyophilization)

**Figure 3:**
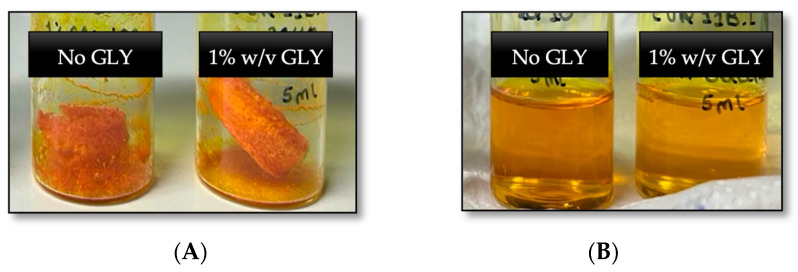
(**A**) CUR-loaded polymeric micelles visual aspect after lyophilization with 1% GLY and with no GLY; (**B**) Lyophilized CUR-loaded polymeric micelles with 1% GLY and with no GLY, visual aspect after re-constitution with water/EtOH mixture, followed by co-solvent evaporation.

The previous unsatisfactory results led us to follow a different approach. Considering that the hydration of the lyophilized powder seemed to be the problem, we tested EtOH as a co-solvent to re-dissolve the freeze-dried powder. Aware that evaporating an EtOH/water mixture under vacuum can be a complex process aggravated by the presence of surfactants, the pressure was lowered progressively, and the final volume was measured and restored, when applicable, to mitigate sample loss. The results were clear solutions with minimal sample loss. Figure 3 illustrates an elegant and non-collapsed cake in samples lyophilized with 1% GLY. 

By comparing the measured average size and PDI before and after freeze-drying, the protective role of GLY in minimizing particle aggregation/fusion becomes clear, evidenced by the inferior size increase in treated samples (Table 4). Regarding CUR content, the amount of entrapped cargo was consistently lower after lyophilization. However, the retention rate of curcumin (RC%) of CUR, highlighted in Table 4, was less accentuated in lyophilized PMs containing 1% GLY compared to the group with no cryoprotector (95.73% and 78.45%, respectively). From these results, it can be concluded that stress associated with lyophilization was reduced by the presence of GLY.

Overall, adding 1% (*w*/*v*) of GLY to PFT:CUR:B, pre-lyophilization, and re-hydrating with an EtOH/water mixture instead of water alone, seems to be an interesting strategy to mitigate stability problems. Moving forward, lyophilized PFT:CUR:B samples containing 1% of GLY were selected as the optimized formulation to be used in future assays.

#### 2.2.3. In Vitro Drug Release

**Figure 4:**
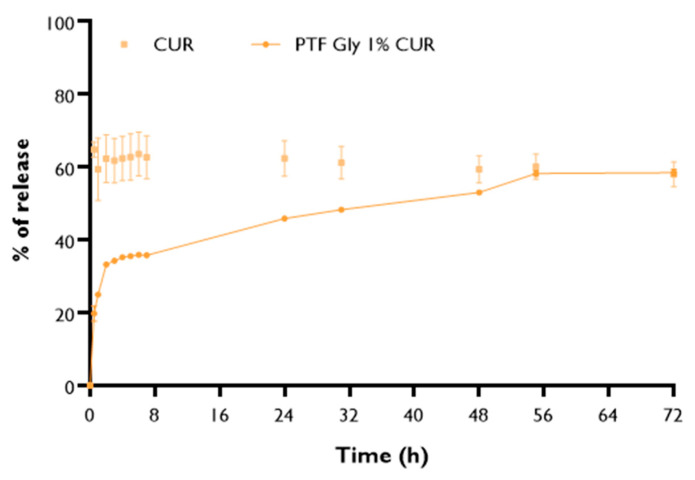
In vitro release profiles of lyophilized CUR loaded polymeric micelles (PFT:CUR:B 1% GLY) and of free CUR. Results are expressed as mean ± SEM (n = 3).

The in vitro release of CUR from PMs was investigated by the dialysis method with 5% sodium dodecyl sulfate (SDS) solution in phosphate-buffered saline (PBS) (pH 7.4) as a release medium for 72 h. As shown in Figure 4, both the PM formulations and the free drug displayed a typical initial burst release in the first 30 min. This is favorable for rapidly attaining the effective therapeutic concentration [74]. As expected, this burst was more significant in free CUR. In fact, only approximately 30% of CUR was released from the PMs within the first 7 h, while more than 60% of the free CUR was released during the same period. The PM carrier can not only solubilize the poorly soluble CUR, but also sustain CUR release for more than 72 h. The cumulative release rate of CUR from the PMs after 72 h was approximately 50%. A sustained release could minimize the exposure of healthy tissues and enhance the accumulation of anti-cancer drugs in tumor regions [75]. The sustained-release characteristics are due to hydrophobic interactions between the encapsulated hydrophobic CUR and the micellar core [20,49]. The initial decrease in free CUR could be due to the molecule’s unstable behavior, as reported by others [76]. 

Lastly, release profiles were fitted with four different mathematical models (zero order, first order, Higuchi, Korsmeyer–Peppas), and the compatibility of the fit was evaluated through the coefficient of determination (R^2^) (Table 5). CUR-loaded PMs follow the Korsmeyer–-Peppas release profile model (power law). This model is frequently used to describe the drug release mechanisms from PMs [74]. 

#### 2.2.4. Critical Micelle Concentration (CMC)

**Figure 5:**
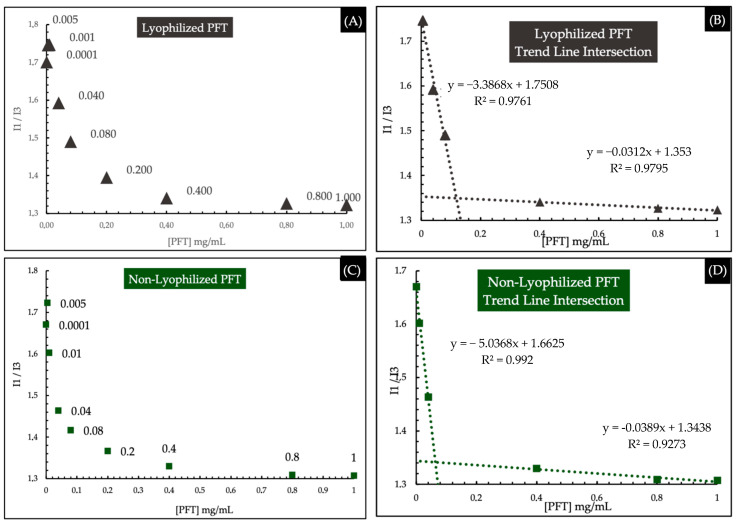
(**A**) The I1/I3 fluorescence intensities ratio (I1/I3) as a function of polymer concentration ([PFT]) for Lyophilized PFT; (**B**) The interception of the trend lines for CMC determination for Lyophilized PFT; (**C**) The I1/I3 fluorescence intensities ratio (I1/I3) as a function of polymer concentration ([PFT]) for Non-Lyophilized PFT; (**D**) The interception of the trend lines for CMC determination Non-Lyophilized PFT.

The CMC value of lyophilized and non-lyophilized PMs was determined by the pyrene fluorescence method. Figure 5 shows the ratio between first and third fluorescence intensities (I1/I3) as a function of polymer concentration ([PFT]) and the interception of the trend lines. The calculated CMC was approximately 0.119 mg/mL and 0.064 mg/mL, for lyophilized and non-lyophilized PMs. The CMC value of TPGS has been reported to be 0.2 mg/mL [49]. A low CMC value is crucial to ensure that even after being diluted by blood dilution (after administration), polymers have the minimum concentration for micelle formation [77,78]. If diluted below the CMC, PMs are unstable and gradually disintegrate [79]. 

Although lyophilization increased the CMC value, pointing to a less stable system, the storage stability of PMs was better after lyophilization. The lyophilization process and the addiction of GLY could influence interactions responsible for micelle formation, affecting the CMC value.

#### 2.2.5. Storage Stability

**Figure 6:**
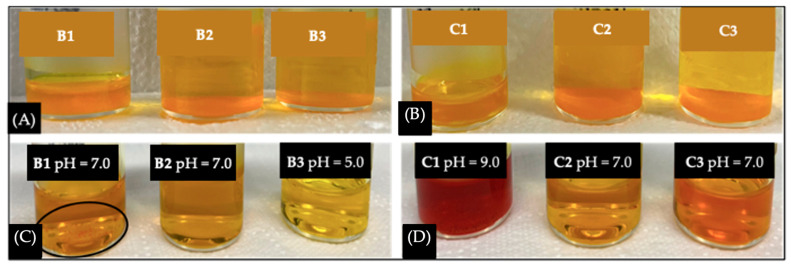
(**A**) Non-lyophilized PFT:CUR:B (B1, B2 and B3) after 30 days at RT; (**B**) Non-lyophilized PFT:CUR:B (C1, C2 and C3) after 30 days at RT; (**C**) PFT:CUR:B samples (B1, B2, and B3) were lyophilized in absence of GLY and then evaluated after 30 days at RT; (**D**) PFT:CUR:B (C1, C2 and C3) were lyophilized with 1% of GLY and then evaluated after 30 days at RT.

To further confirm the hypothesis discussed in Section 2.2, non-lyophilized PMs were visually analyzed after 30 days. All samples had a considerable amount of undissolved CUR deposited at the bottom of each vial, illustrated in Figure 6A, whereas a significant sediment was not visible in PMs after lyophilization, the reason being that lyophilization can extend PM stability over a 30-day period. However, this is not so linear. 

As summarized in Table 6, a progressive aggregation in PMs with no GLY is responsible for the exponential increase in size, 21.387 to 1093.063 nm, and the increase in PDI values at the 30-day end mark. Also, the presence of visible CUR aggregates in Figure 6B, sample B1, further confirms any suspicions that CUR is beginning to precipitate. This can happen due to particle aggregation and structural instability, leading to PM content leakage. Regarding CUR quantification in PMs untreated with GLY, a combination of particle fusion and drug precipitation is responsible for clouding the actual values of EE% and DL% after the 30 days, since there was an increase in CUR concentration, from 1.380 ± 0.253 mg/mL to 1.406 ± 0.877 mg/mL. In other words, these measurements did not represent the content remaining inside the PMs because some of the precipitated CUR might have been mistakenly quantified, and, due to aggregation, the micelle population was not homogeneous.

On the other hand, samples treated with GLY were less prone to aggregation after 15 and 30 days. The GLY group had a more constant rise in size and PDI (Table 6). Even though these results indicated a more stable system, the quantification results did not follow the same trend. Contrary to untreated samples, after 15 and 30 days, CUR concentration in treated samples declined from 1.772 ± 0.168 mg/mL to 1.378 ± 0.369 mg/mL and 1.271 ± 0.068 mg/mL, respectively (Table 6).

An increase in pH in one of the samples (C1) could cause the sharp decrease in CUR concentration and the evident color change [57]. This may have happened because at a pH above neutral CUR molecules are unstable and suffer rapid hydrolytic degradation [56,58]. On the other hand, a pH increase has been linked to instability, contributing to particle aggregation and degradation [58]. This behavior could be related to possible incomplete evaporation of EtOH. In the future, we will consider using co-solvents that are easier to remove by evaporation when in an aqueous solution (e.g., acetone, methanol).

### 2.3. In Vitro Studies of the Optimized Nanosystems—Lyophilized Polymeric Micelles

#### Cell Viability

**Figure 7:**
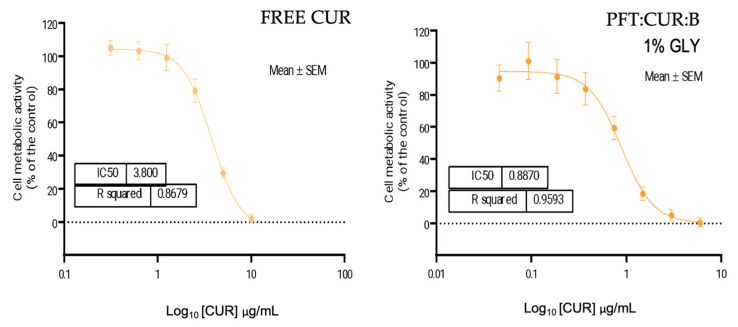
LoVo cells’ metabolic activity after treatment with CUR in DMSO 0.1% (0.313–40 µg/mL of CUR) over 72 h and after treatment with PFT:CUR:B_L 1% GLY (0.046–5.941 µg/mL of CUR) over 72 h. Data are expressed as mean ± standard error of the mean (SEM), (n = 3).

The cytotoxic effects of free CUR and PFT:CUR were evaluated on LoVo cells. After 72 h, a reduction in cell metabolic activity was observed in about 50% when treated with 3.800 and 0.887 μg/mL of CUR, for free CUR and loaded micelles, respectively (Figure 7). Li (2007) reported an approximate half maximal inhibitory concentration (IC_50_) value for free CUR using the same cell line. Based on these results, it is apparent that the encapsulation of CUR in PMs is advantageous in terms of increased cytotoxic effect, compared to free CUR. 

**Figure 8:**
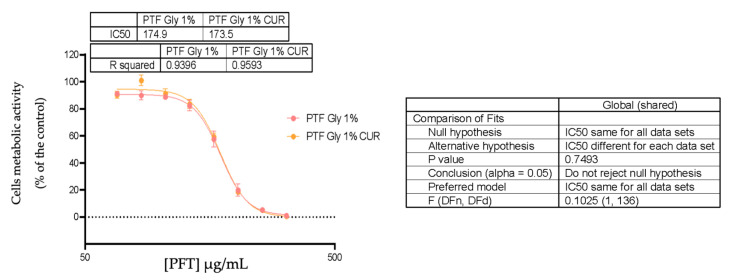
Comparison of LoVo cells’ metabolic activity after treatment with PFT:B_L 1% GLY and PFT:CUR:B_L 1% GLY (67.109–320 µg/mL of polymers) over 72 h.

Despite the existence of a benefit of encapsulating CUR in PMs, it is important to mention that the PFT GLY 1% nanosytem could contribute to this result, as comparing the IC_50_ of PFT:CUR:B_L 1% GLY expressed in polymer concentration ([PFT]) and compared with empty PMs we found these values to be similar (IC_50_ of PFT:CUR = 173.5 μg/mL and IC_50_ of PFT = 174.9 μg/mL). Figure 8 demonstrates a tendential approximation in viability curves between cells treated with loaded and unloaded PMs. More precisely, the polymers contribute to the determined toxicity of loaded PMs, probably due to an insufficient [CUR] inside PFT:CUR compared to the polymer ratio, incomplete drug release after 72 h (approximately 50%), and the potent cytotoxic effect of TPGS. Table 7 compares the different IC_50_ values expressed in [CUR] and [PFT].

A similar conclusion was found by Lee (2015), as a result of the cytotoxic effect of loaded PMs with TPGS [80]. However, encapsulation of the drug inside the systems was still beneficial, due to a selective anticancer effect attributed to TPGS [81]. In future assays, cytotoxicity in cancer cells of both PFT:CUR:B and free TPGS should be compared with normal cells to attest TPGS’s selective antitumor effect and possible cooperative mechanism with CUR. In addition, testing freshly produced PMs would be interesting, as even though these systems are not stable over time, we know they have higher percentages of drug entrapment.

## 3. Discussion

Poor clinical outcomes and limitations of current therapies for CRC create the urge to find innovative treatments that are specific and have fewer adverse reactions [8]. CUR can pose as a safer and more effective alternative for cancer patients’ treatment. However, for CUR to reach therapeutic concentrations in the tumor site, a nano-DDS is necessary [15,82,83]. We proposed the encapsulation of CUR in P123:F127:TPGS PMs as a strategy to overcome its hydrophobic nature, as well as improve its stability and bioavailability for maximum delivery and overall maximum therapeutic effect.

Overall, the findings discussed in Section 2.1 suggest a maximum CUR entrapment, smaller particle size, and neutral ZP when PMs were formed at RT. In fact, avoiding thermal processing could prevent CUR’s degradation, making this process more advantageous. The characteristics of the obtained PMs are also desirable to leverage the EPR effect [53]. Furthermore, it is easier to scale up a manufacturing process conducted at RT. However, eliminating heat from the hydration step could potentially slow down the process.

Unfortunately, the formulations had short storage stability, as physiochemical characterization after 30 days pointed to the occurrence of drug leakage and particle aggregation. Previous studies have connected these signs of instability to free polymer chains [45,49,70]. Hence, lyophilization was sought out to overcome stability issues. However, there were issues in the reconstitution of the freeze-dried powder, caused by the stress of this technique [68,71,72]. Changes in the pre-lyophilized formulation as well as post-lyophilization resulted in minimal drug loss (95.73% of the retention rate of CUR). Lyophilization resulted in a slight increase in CMC; however, the values were still lower than TPGS’s CMC [49]. For this reason, we can anticipate good in vivo stability after being systemically administered. 

PMs gradually release their payloads during blood circulation; however, when the structure is to stable only a relatively small portion of the payload is delivered to the tumor sites, and efficacy in cancer treatment is restricted [52]. In this case, an initial burst followed by a sustained release of CUR is favorable for therapeutic concentrations to be rapidly achieved and maintained over a lengthier period of time [75]. This way, CUR is accumulated in tumor regions and efficacy is not compromised by drug release [74]. 

Stability was evaluated after 1 month in storage. Indeed, the lyophilization process managed to extend PM stability compared to non-lyophilized PMs, as reported by others [73,84,85,86]. On the other hand, the presence of GLY slowed down collision and particle aggregation. A previous study has found similar conclusions [71]. Nonetheless, PFT:CUR:B_L 1% GLY still faced some issues, like pH changes, that contributed to PM aggregation and drug leakage. Many authors have faced similar issues [56,58]. Regarding in vitro results, free CUR exhibited a far less toxic effect than when encapsulated in PMs. However, we could not exclude the hypothesis that the heightened cytotoxic effect of CUR when encapsulated in PMs was, in fact, due to the polymer’s toxicity [87]. In conclusion, the present study found the following strategies to be incremental steps toward achieving an optimized PM loaded with CUR:

1. Improving CUR entrapment by removing thermal processing in micelle production, more specifically, in the hydration step (Section 2.1.);
2. Optimization of the lyophilization protocol by adding 1% GLY to the formulation, and post-lyophilization, reconstitute the lyophilized powder using a water/EtOH mixture (Section 2.2).

Future refinements should focus on strengthening the hydrophobic interaction between CUR and the core-forming polymers to improve CUR retention and stability. A possible strategy would be to increase the TPGS concentration, with good results in other studies [45,70]. Another discussed strategy is the incorporation of CUR/CD [63,64,88]. In terms of lyophilization, other co-solvents (e.g., acetone, methanol) should be tested for re-constitution. This is based on the complexity of evaporating a water/EtOH mixture, which when incomplete can destabilize formulations. On the other hand, it would be interesting to compare long-term stability when formulations are stored as a freeze-dried powder, as many have reported good results before by following this strategy [68,84,89], as freeze-dried products are generally more stable than nanoparticles suspended in an aqueous medium. This is because of the hydrolytic action of water on the polymer matrix that could lead to several problems such as drug leakage or even result in different release profiles [68,73,84]. In addition, aqueous solutions are also prone to the development and growth of microorganisms [73]. 

Lastly, cell viability results should be compared with assays conducted in a healthy cell line, to attest the selective toxic effect of TPGS, for a better interpretation of the results [49]. Also, repeating this assay with freshly produced PMs would be interesting, since they exhibited the highest percentage of CUR entrapment. Another possibility would be to evaluate cell viability after 72 h to allow more CUR to be released from the system. Therefore, we could better differentiate the toxicity increase when CUR is added to the systems from the polymers’ toxicity.

Generally, there are still challenges to overcome before regarding CUR-loaded PMs as a viable candidate for treatment applications in CRC. Hence, developing research in this field constitutes a trail to complete, in order to provide translational clinical applications of CUR-loaded PMs closer to a much-needed therapeutic application for CRC.

## 4. Materials and Methods

### 4.1. Materials

Synthetic curcumin (purity > 97%) was supplied by ©TCI AMERICA (Portland, OR, USA). Pluronic F127 (Mw of 12,600 Da), Pluronic P123 (Mw of 5750 Da), and D-ɑ-tocopheryl polyethylene glycol succinate (Vitamin E TPGS or TPGS) were purchased from SIGMA-ALDRICH^®^ (St. Louis, MO, USA). Acetonitrile (ACN) was from Carlo Erba (Val de Reuil, France). Ethanol (EtOH), absolute, ~98% (GC), was from Honeywell (Saint Germain En Laye, France). Sodium dodecyl sulfate (SDS) 98.5% (GC), pyrene, glycine (GLY), mannitol, sucrose, sorbitol, and sodium chloride were purchased from SIGMA-ALDRICH^®^ (St. Louis, MO, USA). Sodium hydrogen phosphate and potassium dihydrogen phosphate were from MERK^®^ (Oakville, ON, Canada). SnakeSkin™ dialysis tubing (3.5K MWCO, 22 mm I.D.) was supplied by Thermo Fisher Scientific (Austin, TX, USA). Acetone was from Honeywell (France). Dulbecco’s modified Eagle F-12K medium (DMEM) (L0090) was purchased from Biowest^®^ (Nuaillé, France). Dimethyl sulfoxide (DMSO) was acquired from Honeywell (Tokyo, Japan). LoVo cell line (ATCC CCL-229) was provided by ATCC (Rockville, CT, USA). Trypsin and resazurin were provided by SIGMA-ALDRICH (St. Louis, MO, USA and Buchs, Switzerland).

### 4.2. Methods

#### 4.2.1. Development of P123:F127:TPGS Polymeric Micelles

**Figure 9:**
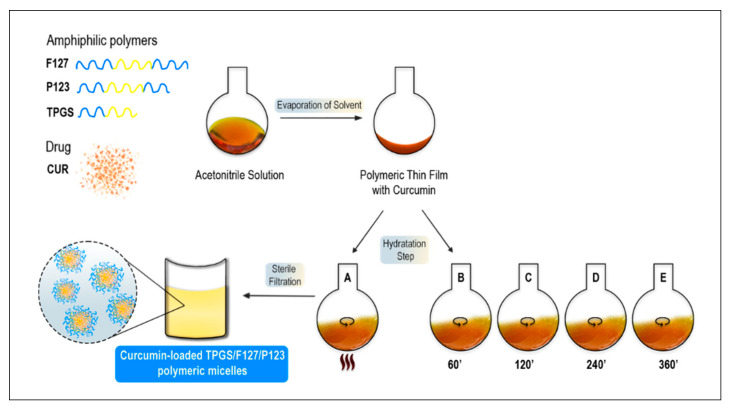
Schematic illustration of the process of developing PFT:CUR with different hydration conditions. The thin-film process includes a step of solvent evaporation, followed by hydratation and sterile filtration.

PMs were prepared by a previously described thin-film hydration method, illustrated in Figure 9 [49,90]. The first step consisted of weighing, approximately, 40 mg of CUR and the polymers (P123, F127, and TPGS) in a 2:1:1 weight ratio, respectively. Next, the polymers were dissolved in 10 mL of an organic solvent (e.g., acetonitrile (ACN)). After being completely solubilized, the solutions were transferred to a round-bottom flask with the active compound and magnetic stirring was used to help to dissolve the solution. Afterwards, the organic solvent was removed via evaporation at 50 °C, under reduced pressure (70–110 mbar), for 60 min. To ensure the complete elimination of the solvent, the formulation was placed in a desiccator under vacuum overnight at RT. The following day, the thin film previously formed was hydrated with 6 mL of ultrapure H_2_O (pH = 7). Lastly, the mixture was filtered through a 0.45 μm cellulose acetate filter to remove the excess of undissolved CUR not encapsulated inside the PMs.

In a quest to achieve maximum CUR entrapment, PMs were prepared using distinct mixing conditions regarding the hydration step: A—the mixture was kept in a 60 °C bath with magnetic stirring for 60 min; B—the mixture was magnetic-stirred for 60 min at room temperature (RT); C—the mixture was magnetic-stirred for 120 min at RT; D—the mixture was magnetic-stirred for 240 min at RT; E—the mixture was magnetic-stirred for 360 min at RT (Figure 9).

#### 4.2.2. Physicochemical Characterization of P123: F127: TPGS Polymeric Micelles

##### Particle Size, Polydispersity Index, and Zeta Potential

Dynamic light scattering (DLS) and electrophoretic light scattering (ELS) were employed to assess the particle hydrodynamic diameter, and the PDI, as well as ZP, respectively, using a Zetasizer Nano-ZS (Malvern Instruments Inc., Worcestershire, UK). All readings of each sample were performed in triplicate at 25 °C. For the average particle diameter and PDI, the samples were transferred to a polystyrene latex cell and were measured at a 173° angle of detection. The surface charge was investigated under the effect of an electric field in a folded capillary cell.

##### Quantification of Curcumin by UV—vis Spectroscopy

The concentration of CUR in each sample was determined by UV—vis spectroscopy according to a previously described method [87]. The absorbance was, respectively, recorded with a double-beam UV–VIS spectrophotometer (Shimadzu UV-1800 UV/Visible Scanning Spectrophotometer), and the spectra were analyzed using UVProbe^®^ software https://www.shimadzu.com/an/products/molecular-spectroscopy/uv-vis/uv-vis-nir-spectroscopy-software/uvprobe/index.html (accessed on 1 July 2024). First, the UV—vis spectra (200–700 nm) of empty and loaded PMs were obtained to make sure the micelle components did not interfere with the analyte maximum absorbance (430 nm) (Figure S1) [49,87]. Previously to absorbance readings, sample preparation was necessary to adjust the absorbance to fit the equipment quantification limit, as well as to disrupt the NP structure. On that note, samples were diluted in absolute EtOH ([EtOH]~99.5% *v*/*v*) in a 1:10 ratio. The resulting product was then diluted 100 times in a prepared EtOH solution ([EtOH] 90% *v*/*v*), resulting in a 1:1000 dilution of the initial product. Finally, the absorbances of a series of standards across a range of concentrations of CUR dissolved in EtOH were fitted in a calibration curve (n = 4) (Figure S2), and the resulting linear regression was used to extrapolate the concentration of the active compound.

##### Drug Loading (DL%) and Encapsulation Efficiency (EE%) Analysis by UV—vis Spectroscopy

Hydrophobic interactions between the active compound and the micellar core determine the degree of encapsulation [91]. The amount of entrapped CUR inside the prepared PMs was obtained through the previously described UV—vis spectroscopy method (Section Quantification of Curcumin by UV—vis Spectroscopy). Briefly, the samples were diluted in a specific volume of EtOH (1:1000) to cause breakage of the micelles and release of the CUR and, next, the absorbance was read at a defined wavelength (430 nm). The extracted result was then converted to a CUR concentration by fitting this value in the previously obtained equation (y = 0.1566x + 0.0058; R^2^ = 0.9997) (Figure S2). Once the CUR concentration in the total volume of the PM solution is known, the DL% and EE% were determined by the following equations:

$$\mathrm{DL}\;\left(\%\right)=\frac{\mathrm{weight}\;\mathrm{of}\;\mathrm{CUR}\;\mathrm{in}\;\mathrm{PFT}:\mathrm{CUR}}{\mathrm{weight}\;\mathrm{of}\;\mathrm{PFT}:\mathrm{CUR}}\times \;100\%\;\mathrm{EE}\;\left(\%\right)=\frac{\mathrm{weight}\;\mathrm{of}\;\mathrm{CUR}\;\mathrm{in}\;\mathrm{PFT}:\mathrm{CUR}}{\mathrm{weight}\;\mathrm{of}\;\mathrm{CUR}\;\mathrm{fed}\;\mathrm{initially}}\times \;100\%$$

##### Storage Stability

According to EMA’s reflection paper on block copolymer micelle medicinal product development, stability studies should address the physical and chemical attributes such as mean size, aggregation, zeta, and stability of the active substance and block copolymer. Therefore, to study the storage stability of the drug-loaded polymeric micelles, freshly prepared samples were incubated at RT protected from light for 30 days. At the 30-day end mark, the size distribution and ZP were measured and compared with freshly prepared samples (0 days). In parallel, the percentage of remaining CUR was determined in order to evaluate possible drug leakage.

##### Lyophilization of P123:F127:TPGS Polymeric Micelles

**Figure 10:**
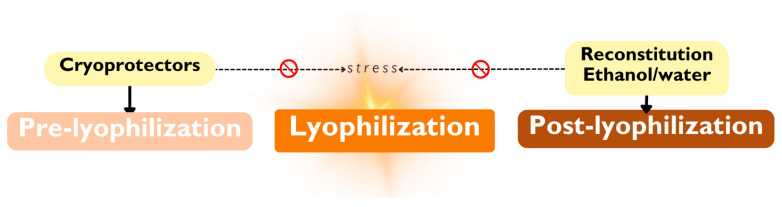
Schematic representation of implemented approaches before and after lyophilization to mitigate stress.

PMs are often lyophilized to overcome instability during long-term storage [73,84]. However, when this process is not specific to the formulation it can induce stress, resulting in physical instability, e.g., aggregation, and drug leakage [68]. The following section will explore two distinct approaches to mitigate stress during lyophilization. The first approach addresses the pre-lyophilization stage, while the second focuses on the post-lyophilization product (Figure 10). Prior to freeze-drying, protector excipients can be added to preserve physicochemical properties. GLY 1% *w*/*v* was added to both empty and loaded PMs. Next, samples were submitted to lyophilization for 24 h. 

Parallelly, we followed a post-lyophilization approach, where the freeze-dried samples (with and without 1% GLY) were then re-suspended in the original volume of water with the help of an equivalent amount of a co-solvent, EtOH 50% (*v*/*v*). Then, the organic solvent was evaporated by gradually reducing pressure. The final volume was measured to study the loss of “water volume” during evaporation.

##### In Vitro Drug Release

Drug release studies were performed to predict the release kinetics of free CUR and PF T:CUR: B_L 1% GLY. Samples were prepared in triplicate (n = 3). A dialysis assay was employed, because the membrane size cut-off (22 mm I.D.) prevents the passage of the nanocarriers, only allowing the diffusion of its content [91,92]. Afterwards, the release kinetics of CUR was investigated using SDS 5% (*w*/*v*) dissolved in PBS (pH 7.4) as the release medium (Table S3), adapted from a previously described method [41]. The tightly sealed dialysis bag was immersed in 80 mL of receptor medium, to maintain sink condition [93]. The experiment was performed under continuous magnetic stirring at 37 ± 0.5 °C. At predetermined time intervals, 2 mL of the release medium was withdrawn and immediately replaced with an equal volume of the release medium. The amount of CUR in the released medium was quantified by UV—vis spectroscopy and extrapolated from a developed calibration curve of standard [CUR] dissolved in the release medium (y = 0.2025x − 0.0016; R^2^ = 0.9998) (n = 3) (Figure S3). In order to determine the cumulative drug released (%) at each time stamp, the previously collected samples were considered. Results are expressed as mean ± SEM (n = 3).

##### Critical Micelle Concentration (CMC)

The CMC can be defined as the minimum concentration of polymer required to form micelles [67,78]. In other words, there is an insufficient number of chains to self-assemble at low polymer concentrations. It is important to determine the CMC of lyophilized and non-lyophilized PMs, since this parameter affects the system’s thermodynamic and kinetic stability, ensuring the cargo is not prematurely released [61,91]. Ideally, a low CMC is desired to avoid PMs dissembling after undergoing severe dilution in the biological environment. Therefore, CMC was determined using a pyrene fluorescent probe [94]. Briefly, the fluorescence turn-on probe is incorporated into the hydrophobic PMs, translating into an increase in emission and confirming micelle formation. For this, a series of polymer solutions at concentrations ranging from 0.0001 to 1 mg/mL were prepared. In parallel, a proper volume of a pyrene stock solution in acetone was pipetted into empty vials and allowed to evaporate at room temperature. Then, a proper volume of each polymer solution was added to each vial to achieve a final concentration of pyrene of 6.0 × 10^−7^ M, and the solutions were left to mix in the dark overnight. After the incubation period, the emission spectra (350–450 nm) of each sample were recorded at a fixed excitation wavelength of 335 nm, using a fluorescence spectrophotometer (Jasco, FP-8200, W. Yorkshire, UK). The intensity ratio of the first peak (I1, 372–374 nm) and the third peak (I3, 382–384 nm) were plotted against the polymer’s concentration ([PFT]).

#### 4.2.3. In Vitro Studies of the Optimized Nanosystem 

Lyophilized PMs with 1% GLY were chosen to evaluate the performance of the system in vitro. This choice was based on the acceptable stability and favorable physiochemical properties previously reported. In addition, empty PMs were used as a control.

##### Cell Culture

Cell culture studies were conducted using LoVo (ATCC CCL-229) cells isolated from a cancer patient with grade IV Dukes C colorectal cancer [95]. In brief, LoVo cells were cultivated in a Dulbecco’s modified Eagle F-12K medium (DMEM) supplemented with 10% fetal bovine serum (FBS) and 1% penicillin/streptomycin and 1% L-glutamine. The cells were maintained in culture at 37 °C in a 5% CO_2_ humidified atmosphere. Cells were plated with 80% of confluence to exclude the negative impact of an overly high confluency on proliferative potential. The cells were used between passages 8–10.

##### Cell Viability 

Cells were seeded in 96-well plates at a density of 5000 cells/mL and allowed to attach during 24 h. After 24 h, the medium was replaced, and the cells were incubated for 72 h with free CUR, PFT:B_L 1% GLY, and PFT:CUR:B_L 1% GLY, at known CUR and polymer concentrations. Before that, the prepared micelle stocks were filtered (Sterile Syringe Filter 22 μm) and the CUR present in the formulation was quantified through a previously described UV—vis technique (Section 4.2.2). After incubation for 72 h, the medium was removed and replaced with 100 μL of growth medium containing 1% resazurin (1 mg/mL) and incubated for two hours. Cytotoxicity was evaluated using the Alamar Blue assay (resazurin reduction assay) [96]. Shortly, viable cells can reduce resazurin into resorufin, a pink and fluorescent product, that can be quantified by measuring a change in absorbance at a wavelength of 570 nm (A570) and 600 nm (A600) in a microplate reader. Medium without cells with 1% resazurin was used as a negative control (C^−^), and untreated cells as a positive control (C^+^). The percentage of cell metabolic activity was calculated using the following equation:

$$\mathrm{Experimental}\;\mathrm{Value}\;\;\;\;\;{\mathrm{Cl}}^{-}\;\%\;\mathrm{Cells}\;\mathrm{Metabolic}\;\mathrm{Activity}=\frac{(A570-A600)-(A570-A600)}{(A570-A600)-(A570-A600)}\;\times \;100\%\;{\mathrm{Cl}}^{+}\;\;\;\;\;\;\;{\mathrm{Cl}}^{-}$$

### 4.3. Data Analysis

The data collected are expressed as mean ± standard deviation (SD) from at least three measurements (n = 3). GraphPad Prism 8 was used to plot dose response curves and to perform the statical analysis. 

## 5. Conclusions

Noticeable differences in the percentage of entrapped CUR between the groups of PMs hydrated at RT (PFT:CUR:B, C, D, and E) and in a 60 °C water bath (PFT:CUR:A) were observed. Concerning storage stability, lyophilization has proven to be a promising strategy to extend shelf-life. Nonetheless, better strategies for optimal stability should be explored. Finally, the potent cytotoxic effect of PFT:CUR:B_L 1% GLY was mainly attributed to the system composition. Although the in vitro studies did not report a clear advantage on encapsulating CUR inside PMs, we know that there are benefits in loading CUR inside PMs, supported by bioavailability and solubility increase. 

## Figures and Tables

**Table 1:** Pluronic’s compositions and properties. Adapted from [36,38,39,45].

	PEOa—PPOb—PEOa	Mw ^1^	Average n° PEO Units ^2^	Average n° PPO Units ^3^	CMC ^4^	HLB ^5^	Applications
P123	PEO_20_—PPO_70_—PEO_20_	5750	39.2	69.4	0.0253	7–9	Inhibition of multidrug-resistance Drug delivery
F127	PEO_101_—PPO_56_—PEO_101_	12,600	200.4	65.2	0.0353	18–23	Provide stability to NPs Long circulating particles Low release gels Tissue engineering
TPGS	-	1513	-	-	0.02	13	Solubilizer of poorly water soluble drugs Enhancer of drug permeability by P-glycoprotein efflux inhibition Stabilizer of amorphous drug dispersion

^1^ Average molecular weight (Mw) in g/mol. ^2,3^ The average numbers of PEO and PPO units per polymer calculated using the average molecular weights. ^4^ Critical micelle concentration (CMC) in mg/mL at 25 °C determined by pyrene probe technique. ^5^ Hydrophilic−lipophilic-balance (HBL) values determined by manufacturer.

**Table 2:** The physicochemical characterization of filtered polymeric micelles, PFTs, and PFT:CUR, prepared under two distinct hydration conditions (A and B) (n = 3).

Hydration Conditions	A		B	
Filtered Sample	**PFT**	**PFT:CUR**	**PFT**	**PFT:CUR**
Size (nm)	22.5 ± 1.2	22.9 ± 3.0	16.2 ± 0.9	15.9 ± 0.3
PDI	0.401 ± 0.042	0.475 ± 0.067	0.201 ± 0.065	0.251 ± 0.103
Zeta (mV)	−4.2 ± 0.8	−2.4 ± 1.9	−1.0 ± 1.8	−1.5 ± 1.9
EE %	-	18.197 ± 3.452	-	35.083 ± 1.144
DL %	-	1.880 ± 0.317	-	3.217 ± 0.091
[CUR] mg/mL		1.224 ± 0.224		2.265 ± 0.194

**Table 3:** The physicochemical characterization of CUR-loaded polymeric micelles (PFT:CUR), prepared under distinct hydration conditions (A, B, C, D, and E), before and after 30 days in storage (n = 3).

Filtered Sample	PFT:CUR:A	PFT:CUR:B	PFT:CUR:C	PFT:CUR:D	PFT:CUR:E
Size (nm)	22.9 ± 3.0	15.9 ± 0.3	17.3 ± 2.9	15.3 ± 0.6	15.9 ± 0.4
30 days	51.0 ± 13.4	25.7 ± 7.3	252.0 ± 25.0	113.8 ± 13.8	612.9 ± 15.8
PDI	0.475 ± 0.067	0.251 ± 0.103	0.204 ± 0.064	0.144 ± 0.062	0.191 ± 0.025
30 days	0.519 ± 0.025	0.319 ± 0.092	0.475 ± 0.475	0.576 ± 0.369	0.884 ± 0.148
Zeta (mV)	−2.4 ± 1.9	−1.5 ± 1.9	−2.6 ± 1.3	−0.2 ± 0.7	−0.8 ± 1.2
30 days	−4.6 ± 7.0	−2.1 ± 3.2	−1.7 ± 0.8	−1.4 ± 1.2	−0.6 ± 1.1
EE %	18.197 ± 3.452	35.083 ± 1.144	29.153 ± 2.276	27.360 ± 5.056	24.837 ± 2.106
30 days	10.690 ± 3.714	20.957 ± 5.309	19.350 ± 4.966	24.363 ± 4.952	16.497 ± 4.233
DL %	1.880 ± 0.317	3.217 ± 0.091	2.590 ± 0.320	2.553 ± 0.305	2.273 ± 0.197
30 days	0.975 ± 0.995	1.906 ± 0.481	1.770 ± 0.450	2.230 ± 0.463	1.507 ± 0.387
[CUR] mg/mL	1.224 ± 0.224	2.265 ± 0.194	1.965 ± 0.164	1.979 ± 0.337	1.522 ± 0.202
30 days	0.896 ± 0.896	1.410 ± 0.357	1.303 ± 0.331	1.644 ± 0.339	1.131 ± 0.301
Remaining CUR%	73.20%	62.25%	66.31%	83.07%	74.31%

**Table 4:** (**A**) Physicochemical properties of loaded polymeric micelles (PFT:CUR:B) before and after lyophilization in the absence of a cryoprotector. (**B**) Physicochemical properties of loaded polymeric micelles (PFT:CUR:B) before and after lyophilization in the presence of 1% (*w*/*v*) of GLY. (n = 3).

**(A)**	**Size (nm)**	**PDI**	**Zeta (mV)**	**EE (%)**	**DL (%)**	**[CUR] mg/mL**
Before Lyophilization	16.9 ± 1.4	0.270 ± 0.072	0.6 ± 0.9	25.825 ± 2.213	2.377 ± 0.197	1.759 ± 0.142
After Lyophilization No GLY	21.4 ± 3.4	0.256 ± 0.011	−3.6 ± 1.4	20.277 ± 3.842	1.702 ± 0.280	1.380 ± 0.253
						78.45%
**(B)**	**Size (nm)**	**PDI**	**Zeta (mV)**	**EE (%)**	**DL (%)**	**[CUR] mg/mL**
Before Lyophilization	17.0 ± 0.8	0.264 ± 0.028	−0.1 ± 0.6	27.082 ± 1.857	2.504 ± 0.143	1.851 ± 0.108
After Lyophilization 1% GLY	19.3 ± 3.7	0.224 ± 0.078	−1.8 ± 0.6	25.926 ± 2.512	2.398 ± 0.228	1.772 ± 0.168
						95.73%

**Table 5:** Drug release data of free CUR and CUR-loaded micelles fitted into four different mathematical models (zero order, first order, Higuchi, Korsmeyer–Peppas). Abbreviations—K: slope; b: Y-intersections; R^2^: coefficient of determination.

		Free CUR	Micelles
Zero Order	K	0.9657	0.00872757
	b	−0.7111	26.7103853
	R^2^	0.9937	0.65993668
Frist Order	K/2.303	0.0001	8.04 × 10^−5^
	log Q0	1.4780	1.47796768
	R^2^	0.7272	0.7272309
Higuchi	K	1.0086	0.65853923
	b	29.0437	19.0791274
	R^2^	0.8234	0.82289694
Korsmeyer–PEPPAS	K	0.1971	16.6820184
	b	1.2313	−5.309242
	R^2^	0.9593	0.96565865

**Table 6:** (**A**) Size, PDI, and zeta of lyophilized CUR-loaded polymeric micelles in the absence of a cryoprotector (without GLY) and with GLY (1%) at 0, 15, and 30 days (n = 3); (**B**) EE%, DL% and [CUR] mg/mL of lyophilized CUR-loaded polymeric micelles in the absence of a cryoprotector (without GLY) and with GLY (1%) at 0, 15, and 30 days (n = 3).

**(A)**	**Size (nm)**			**PDI**			**Zeta (mV)**		
	**0 Days**	**15 Days**	**30 Days**	**15 Days**	**30 Days**	**30 Days**	**0 Days**	**15 Days**	**30 Days**
No GLY	21.4 ± 3.4	77.3 ± 0.1	1093.1 ± 850.0	0.256 ± 0.011	0.239 ± 0.006	0.473 ± 0.429	−3.6 ± 1.4	−4.7 ± 4.6	−4.5 ± 4.5
1% GLY	19.3 ± 3.7	33.4 ± 3.4	37.9 ± 1.5	0.224 ± 0.078	0.378 ± 0.182	0.386 ± 0.259	−1.8 ± 0.6	−4.2 ± 2.8	−4.5 ± 1.7
**(B)**	**EE (%)**			**DL (%)**			**[CUR] mg/mL**		
	**0 Days**	**15 Days**	**30 Days**	**15 Days**	**0 Days**	**15 Days**	**30 Days **	**15 Days**	**0 Days**
No GLY	20.277 ± 3.842	18.299 ± 3.208	20.616 ± 12.791	1.702 ± 0.280	1.684 ± 0.290	1.899 ± 1.181	1.380 ± 0.253	1.246 ± 0.212	1.406 ± 0.877
1% GLY	25.926 ± 2.512	20.204 ± 5.636	21.913 ± 6.427	2.398 ± 0.228	1.863 ± 0.497	1.719 ± 0.093	1.772 ± 0.168	1.378 ± 0.369	1.271 ± 0.068

**Table 7:** IC_50_ values of free CUR, PFT:CUR:B GLY 1%, and PFT:B GLY 1% in LoVo cells over 72 h.

IC_50_	[CUR] µg/mL	[PFT] µg/mL
Free CUR	3.800	-
PFT:CUR:B GLY 1%	0.887	173.5
PFT:B GLY 1%	-	174.9

## Data Availability

Data is contained within the article and Supplementary Material.

## Supplementary Materials

The following supporting information can be downloaded at: https://www.mdpi.com/article/10.3390/ijms25147577/s1. References [6,87,91,92,93,94,95] are cited in the supplementary materials.

## List of Abbreviations

ACN	Acetonitrile
CDs	Cyclodextrins
CMC	Critical micellar concentration
CRC	Colorectal cancer
CUR	Curcumin
DL %	Drug loading percentage
EE %	Encapsulation efficiency percentage
EtOH	Ethanol
GLY	Glycine
I1/I3	Intensity ratio between first and third peak of fluorescence of pyrene
IC_50_	Half maximal inhibitory concentration
Mw	Molecular weight
nano-DDS	Nanotechnology-based drug delivery system
NPs	Nanoparticles
PBS	Phosphate-buffered saline
PDI	Polydispersity index
PEO	Poly(ethylene oxide)
PFTs	P123:F127:TPGS polymeric micelles
PFT:CUR	CUR-loaded P123:F127:TPGS polymeric micelles
PFT:CUR:B_L 1%GLY	CUR-loaded P123:F127:TPGS lyophilized with 1% of GLY
PMs	Polymeric micelle
PPO	Poly(propylene oxide)
RC	Retention rate of curcumin
RT	Room temperature
SDS	Sodium Dodecyl Sulfate
TPGS	Vitamin E TPGS or D-ɑ-tocopherol polyethylene glycol succinate
UV—vis	Ultraviolet–visible spectroscopy
ZP	Zeta potential
