# Supplementary material for: Development and Characterization of Curcumin-Loaded TPGS/F127/P123 Polymeric Micelles as a Potential Therapy for Colorectal Cancer
Source: Int J Mol Sci. 2024 Jul 10;25(14):7577. doi: 10.3390/ijms25147577 (PMC11276776; doi:10.3390/ijms25147577)
Supplement: Supplementary file 1 [file ijms-25-07577-s001.zip › ijms-3054350-supplementary.pdf]

Supplementary Materials:

2. Results

2.1. Development and Characterization of P123:F127:TPGS Polymeric Micelles

2.1.1. Particle Size, Polydispersity Index and Zeta Potential

Taking into consideration the result of eliminating thermal processing on the micelle's characteristics, the impact of the extended hydration times on PM's physicochemical characterization was assessed by defining new conditions - C (120 minutes); D (240 minutes) and E (360 minutes). As shown in Table S1 a general reduction in polydispersity index (PDI) was observed with the increase of the hydration time, indicating that micelle formation was more uniform. Also, PM hydrated at room temperature (RT) (B, C, D and E) had a neutral ZP and are expected to have longer circulation times [87,92,93]. Despite the more extended mixing periods (from 60 min to 360 min), no significant improvements in the EE% and DL% were observed. Therefore, the increase in the hydration time did not manage to encapsulate more CUR inside the hydrophobic core, this was only achieved by removing heat processing from the hydration step in the development of PM. Overall, the presented results demonstrate that PM hydrated at RT are consistently smaller and have a lower PDI (<0.3). More importantly, eliminating thermal processing remains a key factor for increasing EE% and DL% percentages. In summary, PFT:CUR:B has shown the highest value for EE% and DL%, and a promising compromise between size and ZP (Table S1). Based on this, a hydration time of 60 min at RT was chosen to proceed for the following studies.

Table S1. The physicochemical characterization of filtered polymeric micelles, PFT and PFT:CUR, prepared under three distinct hydration conditions (C, D and E) (n=3).

**Hydration conditions**	**C**		**D**		**E**	
**Filtered Sample**	**PFT**	**PFT:CUR**	**PFT**	**PFT:CUR**	**PFT**	**PFT:CUR**
**Size (nm)**	17.1 ± 3.0	17.3 ± 2.9	16.3 ± 0.5	15.3 ± 0.6	18.0 ± 2.3	15.9 ± 0.4
**PDI**	0.230 ± 0.101	0.204 ± 0.064	0.209 ± 0.031	0.144 ± 0.062	0.259 ± 0.077	0.191 ± 0.025
**Zeta (mV)**	-0.9 ± 0.9	-2.6 ± 1.3	-1.9 ± 1.9	-0.2 ± 0.7	-2.5 ± 3.9	-0.8 ± 1.2
**EE %**	-	29.153 ± 2.276	-	27.360 ± 5.056	-	24.837 ± 2.106
**DL %**	-	2.590 ± 0.320	-	2.553 ± 0.305	-	2.273 ± 0.197
**[CUR] mg/mL**	-	1.965 ± 0.164		1.979 ± 0.337	-	1.522 ± 0.202

Parallelly, loaded PM were prepared following a similar thin-film method, yet in this case, the organic solvent used was ethanol (EtOH). This change in solvent was made with the intention of improving CUR's incorporation inside the hydrophobic core. However, the incorporated CUR rapidly precipitated, deeming this protocol unsuitable. Later we found in literature that ethanol has been shown to overstretch chains of some Pluronic copolymers, like F127, destabilizing interactions responsible for micelle formation [94]. Therefore, contributing to structural instability and drug leakage.

2.1.2. Quantification of Curcumin using UV-Vis Spectroscopy

First, in order to verify if the PFT empty PMs could interfere in the quantification of the active compound spectrum scanning of empty PFT and free CUR in 90% (v/v) of EtOH were performed. As shown in Figure S1 the empty PMs did not reveal absorption peaks in the range of CUR, making

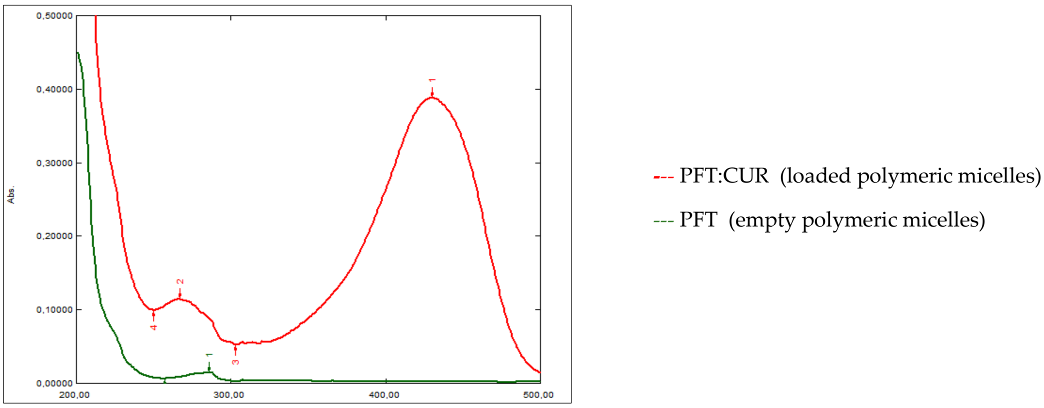

possible the application of this method to quantify CUR.

Figure S1. UV-vis spectra for PFT:CUR (loaded polymeric micelles) in ethanolic solution, and PFT (empty polymeric micelles) in ethanolic solution.

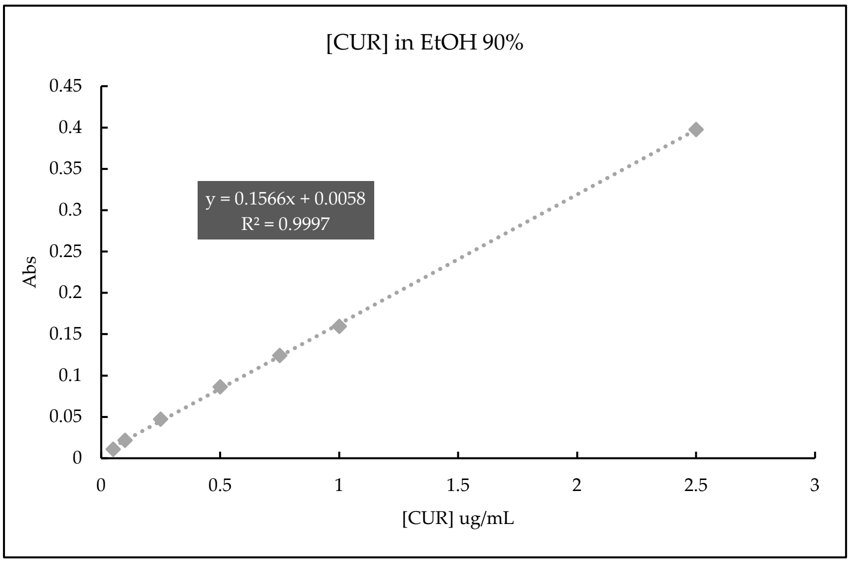

Figure S2. Calibration curve of standards [CUR] dissolved in EtOH (n=4).

2.2. *Development and Characterization of Lyophilized P123:F127:TPGS Polymeric Micelles*

Table S2. The physicochemical characterization of lyophilized CUR-loaded polymeric micelles (PFT:CUR), prepared under distinct hydration conditions (A, B, C, D e E) (n = 3).

	PFT:CUR after centrifugation				
Hydration conditions	**A**	**B**	**C**	**D**	**E**
**Size (nm)**	23.0 ± 6.4	18.3 ± 1.7	*163.3 ± 255.7*	*58.2 ± 36.5*	*130.0 ± 189.3*
**PDI**	0.337 ± 0.138	0.301 ± 0.077	0.372 ± 0.338	0.337 ± 0.139	0.457 ± 0.393
**Zeta (mV)**	-2.3 ± 4.6	-3.7 ± 3.2	-3.4 ± 1.8	-0.9 ± 0.8	-1.4 ± 2.0
**EE (%)**	18.073 ± 0.491	17.341 ± 1.520	21.679 ± 5.938	18.239 ± 5.478	22.880 ± 0.879
**DL (%)**	1.463 ± 0.055	1.719 ± 0.339	1.982 ± 0.727	1.704 ± 0.527	2.090 ± 0.078
**[CUR] mg/mL**	0.973 ± 0.269	1.943 ± 0.404	1.486 ± 0.417	1.292 ± 0.297	1.288 ± 0.296

2.2.3. *In Vitro* Drug Release

Firstly, a pilot experiment was conducted to determine CUR solubility in several mediums, and the appropriate concentration of CUR was determined to use in the drug release assay. CUR was more soluble in Tween-80 10% (0.888 ± 0.068 mg/mL) and SDS 5% (0.435 ± 0.029 mg/mL) (Table S3). Taking it into consideration, free CUR and the optimized lyophilized loaded PM were dissolved in the release medium (SDS 5%) to reach a final [CUR] of 300 μg/mL (below CUR's estimated solubility in SDS 5%).

Table S3. Apparent solubility of CUR (mg/mL) in Tween-80 0.5% (v/v); Tween-80 10% (v/v); PEG-400 30% (v/v); SDS 5% (w/v) in phosphate buffered saline (PBS, pH = 7.4)

	PBS	Tween-80 0,5% (v/v)	Tween-80 10% (v/v)	PEG-400 30% (v/v)	SDS 5% (m/v)
**CUR (mg/mL)**	0.005 ± 0.002	0.086 ± 0.001	0.888 ± 0.068	0.014 ± 0.001	0.435 ±0.029

4.2.2.6 *In Vitro* Drug Release

The design of the *in vitro* drug release assays was based on a preliminary solubility test of CUR solubility in different release mediums. On a basis, the apparent solubility of CUR in several mediums - Tween-80 0.5% (v/v); Tween-80 10% (v/v); PEG-400 30% (v/v); SDS 5% (w/v) in phosphate buffered saline (PBS, pH=7.4) - was investigated using the Flask-shake method [6,91,95]. Beforehand, PBS was prepared using a protocol adapted from the portuguese pharmacopeia [95]. Shortly, CUR (1-2 mg/mL) was added to each medium. The solutions (n=3) were stirred for 24h at 37 ± 0.5ºC. Next, the collected samples were centrifuged to separate the deposit from the saturated solution followed by filtration (nylon filters 0.45 μm). Then, samples were diluted in ethanol and analysed by UV-Vis spectrometry and quantified using a previously determined calibration curve (4.2.2.2).

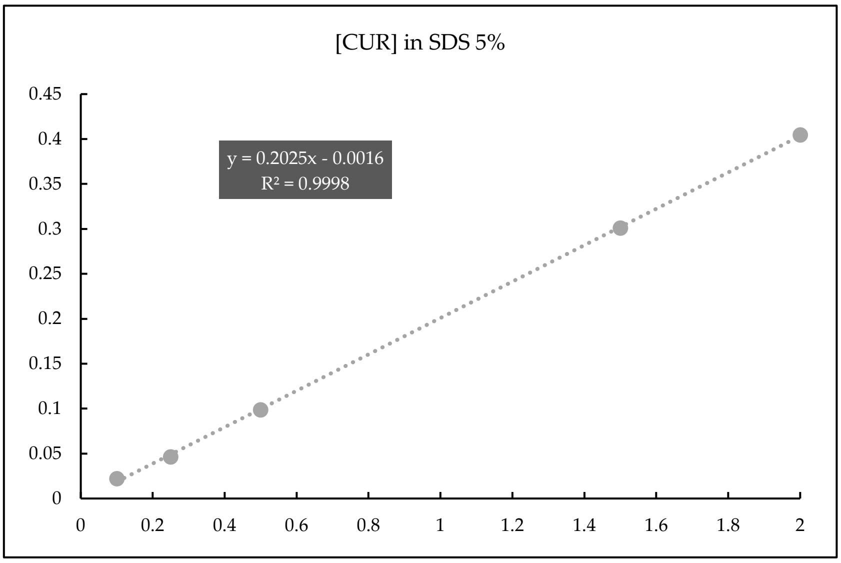

Figure S3. Calibration curve of standards [CUR] dissolved in SDS (n=3).
